# In Vitro Chromatic Performance of Three Presbyopia-Correcting Intraocular Lenses with Different Optical Designs

**DOI:** 10.3390/jcm11051212

**Published:** 2022-02-24

**Authors:** Diego Montagud-Martínez, Vicente Ferrando, Anabel Martínez-Espert, Salvador Garcia-Delpech, Juan A. Monsoriu, Walter D. Furlan

**Affiliations:** 1 Centro de Tecnologías Físicas, Universitat Politècnica de València, 46022 Valencia, Spain; diemonma@upvnet.upv.es (D.M.-M.); viferma1@upv.es (V.F.); jmonsori@fis.upv.es (J.A.M.); 2 Departamento de Óptica y Optometría y Ciencias de la Visión, Universitat de València, 46100 Valencia, Spain; anabelmartinez@aikenval.com; 3 Clínica Aiken, Fundación Aiken, 46004 Valencia, Spain; salvadorgarciadelpech@gmail.com

**Keywords:** presbyopia correcting IOLs, EDOF IOLs, chromatic aberration, refractive segmented IOLs, optical bench

## Abstract

Most of the new premium models of intraocular lenses for presbyopia correction use diffractive optics in their optical design. The presence of multiple foci and the difference of the diffractive efficiency for different wavelengths have a great impact in the lens optical performance. In this context, there is a limited information available for clinicians to understand the optical principles that differentiate each design and their potential influence on clinical outcomes. Optical bench studies with polychromatic light are necessary to solve this limitation. In this work, a custom made optical bench was employed to assess with polychromatic light the through the focus optical quality of three different IOL designs: *trifocal*, *EDOF effect*; *and enhanced monofocal*. By using different and complimentary approaches: images of the USAF test, axial PSFs and TF-MTFs, each design revealed its intrinsic features, which were not previously reported for these IOLs models in a comparative way. It was found that the chromatic aberration plays a very important role in the performance of each IOL. Our results could help clinicians to understand the optical principle of each lens and also provide useful information for choosing the lens that best suits the needs of the individual patient.

## 1. Introduction

As the expectation of good vision at intermediate distances becomes more and more important for the daily life of patients after refractive or cataract sur, new models of intraocular lenses (IOLs) are continuously emerging in the market. Most of these IOLs use diffractive optics in their optical design [1,2,3].

Within diffractive lenses, there are designs that superimpose two independent diffractive bifocal profiles to achieve an extended range of vision with three main focal distances: far, intermediate and near [4]. From a clinical point of view, both the presence of multiple foci and the dependence of the diffractive efficiency of the lens for each wavelength may have a negative effect on the occurrence of dysphotopic effects [5,6]. To minimize these effects, new lenses have been developed that use another concept, touted as extended depth of focus (EDOF). The American Academy of Ophthalmology working group published a consensus statement for these IOLs, consisting of considering EDOF a lens that provides an extension of focus that is at least 0.50 D wider than for a monofocal IOL at a visual acuity (VA) of 0.2 logMAR [7]. Being classified according to a clinical rather than a physical point of view, there are multiple types of IOLs with different optical designs within the EDOF category. In a recent paper, Rampat and Gatinel [3] proposed a nomenclature to classify these lenses. Within this classification are the “EDOF effect IOL” lenses. The diffractive profile of this type of IOLs, such as the *TECNICS Symfony* and the *AT LARA*, has a distinctly different diffractive efficiency for each wavelength and the superposition of foci with different efficiencies generates an extension of focus with white light [8,9,10,11]. Another group of lenses within the EDOF classification [3] is the so-called “enhanced monofocal”, also known as mono-EDOF. This group includes one of the most recent EDOF designs: the *xact Mono-EDOF ME4* lens, in which a diffractive profile of only a few rings is added on a monofocal, aspheric base lens to reduce negative dysphotopic effects and, at the same time, to increase the range of sharp vision. Although there are some clinical studies with this lens [12,13]; its optical performance in vitro has been assessed only with monochromatic light [14,15]. 

In this context, there is still a limited objective information to enable clinicians to understand the optical principles that differentiate each design and their potential impact on clinical outcomes. Thus, it is sometimes difficult for surgeons to really know the main features (advantages and disadvantages) of each design, and often ophthalmic surgeons must rely on advertising and data provided by the manufacturers, who sometimes use confusing terminology to claim advantages over their competitors in the market. Therefore, comparative in vitro tests of different commercial IOLs with polychromatic light could provide important data to fill this gap. In the ISO 11979-2 standard [16], the use of monochromatic green light of wavelength 546 nm (where the human eye has a higher sensitivity [17]) is recommended for the optical testing of multifocal IOLs. However, it is evident that studying the optical performance of IOLs in monochromatic light is insufficient to extrapolate its visual performance. To real-life conditions [10] and optical bench studies with polychromatic light are necessary. Moreover, this aspect is particularly important because it should be taken into account that several of the latest IOL models exploit chromatic diffractive effects in their designs [18,19,20]. Therefore, for an objective study of the performance of an IOL in polychromatic light, it is necessary to test it in an ISO-compliant optical setup, using different wavelengths independently and also in white light. In this sense, obtaining images of the USAF test under white light illumination is a good way to show the through the focus optical quality of a multifocal IOL. However, differences between IOLs optical designs are masked in this process. On the other hand, measurements of the polychromatic point spread functions (PSFs) along the optical axis can be used to make a statement about the quality of an IOL and the axial distribution of the different foci. This would provide an insight into the effect of the superimposition of different wavelengths in the focal range of each lens that would allow an understanding of the causes of the dysphotopic phenomena and its influence on the visual performance of the IOL. Complementarily, the chromatic study of the through-focus modulation transfer function (TF-MTF) would allow us to obtain information on the variation of the contrast of the images provided by the IOL at different vergences with different wavelengths in the visible range.

Considering these facts, the aim of this work is to evaluate comparatively, under the same conditions in custom made optical bench, the chromatic performance of three diffractive IOLs, each one representative of the above mentioned categories (trifocal, EDOF effect and enhanced monofocal IOLs), using different approaches: images of the USAF test, axial PSFs and TF-MTFs. Our purpose is to provide objective evidence to surgeons that allow them to differentiate the characteristics of each lens with their relative advantages and disadvantages.

## 2. Materials and Methods

### 2.1. IOL Designs Studied

The following IOLs were tested: xact Mono-EDOF ME4 (Santen Pharmaceutical Co. Ltd., Osaka, Japan [21]), AT LARA 829MP (Carl Zeiss Meditec, Jena, Germany [22]), and trifocal FineVision POD F (PhysIOL, Liège, Belgium [23]). Table 1 lists the main specifications of the three studied lenses. 

The xact Mono-EDOF ME4 has 4 diffractive rings in the central 3.00 mm of the optical zone on the anterior surface. The posterior surface has an aspheric design. 

The AT LARA 829MP optical design is diffractive and aspheric. It has an aberration-neutral and chromatic aberration-correcting optical design. 

The FineVision POD F is a trifocal IOL, with a diffractive profile composed of two overlapping additions on the anterior face of the lens and an aspheric posterior surface. This design provides a distance refractive focus, and two diffractive foci for intermediate (1.75 D) and near (3.50 D) vision.

### 2.2. Experimental Setup and Metrics

**Figure 1:**
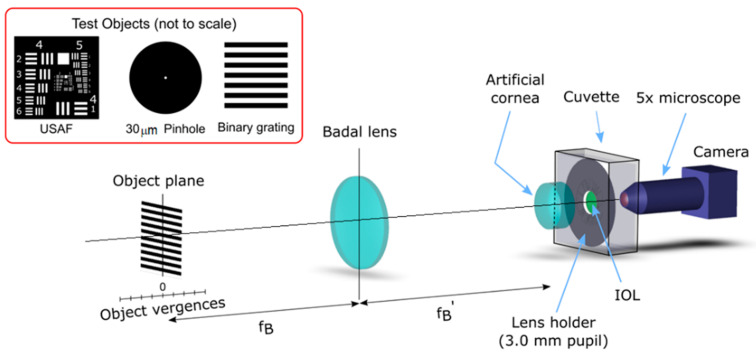
Scheme of the experimental setup.

A custom made optical bench, conceived to accomplish the requirements of the International Organization for Standardization ISO 11979-2, 2014 [16], was employed in this study. Figure 1 shows a schematic representation of the optical setup. It is an image formation system capable of obtaining the focus images of different test objects provided by multifocal IOLs in a model eye. In this way, the PSFs can be registered by using a pinhole object, and the TF-MTF can be measured as the relative contrast of the image of a square grating. A detailed description of the optical setup was provided elsewhere [24].

The model eye consisted of an achromatic lens (Melles Griot LA034 27.8D) [25] with corrected spherical aberration (ISO Model 1 cornea [16]) and a wet cell filled with a saline solution, in which a lens holder with the IOLs under test were placed (see Figure 1). The images provided by the model eye were captured by a CMOS camera (EO-5012C; Edmund Optics, Barrington, NJ, USA) attached to an X5 microscope objective. The camera and the objective were mounted on an XYZ translation stage to precisely adjust the image plane of the virtual retina. In the experiment, a 3.0 mm diameter pupil was employed to avoid the mutual interaction between spherical aberration and LCA [18,19]. Moreover, to isolate the polychromatic optical performance of the IOLs from the residual chromatic aberration generated by the other optical elements in the optical bench, the LCA of the optical bench (without IOL) was measured and subtracted from the experimental TF-MTF curves. 

To study the image formation properties of the IOLs, the 1951 USAF resolution test chart was used first as a test object. Then, the PSFs generated by the IOLs at different vergences were obtained using a 30 µm pinhole as test object. Finally, the TF-MTFs (at 50 lp/mm) at each vergence was calculated from the contrast of the image of a periodic binary grating as a test object (see the inset in Figure 1). Moreover, in this study, the LCA was obtained from the corresponding TF-MTF curves as the difference between the blue (450 nm) and the red (650 nm) focal planes. 

The test objects were mounted on a motorized translation stage (Thorlabs LTS300/M, travel range 300 mm, accuracy ±5 µm) and a Badal lens, was used to provide different vergences in the −2 D to 5 D range. The test objects were illuminated by a collimated beam provided by a Cold White LED (MCWHL5; Thorlabs Inc, Newton, NJ, USA, 6500 K, not shown in the figure) with a bandwidth ranging from 420 nm to 680 nm [26]. The camera’s white balance was set to 6500 K. Moreover, three 10 nm bandwidth filters with central wavelengths at 450 nm, 550 nm, and 650 nm (FBK450-10; FBK550-10; FBK650-10; Thorlabs Inc, Newton, NJ, USA) were employed to obtain the quasi monochromatic images. 

## 3. Results

### 3.1. TF-USAF and PSF Images

**Figure 2:**
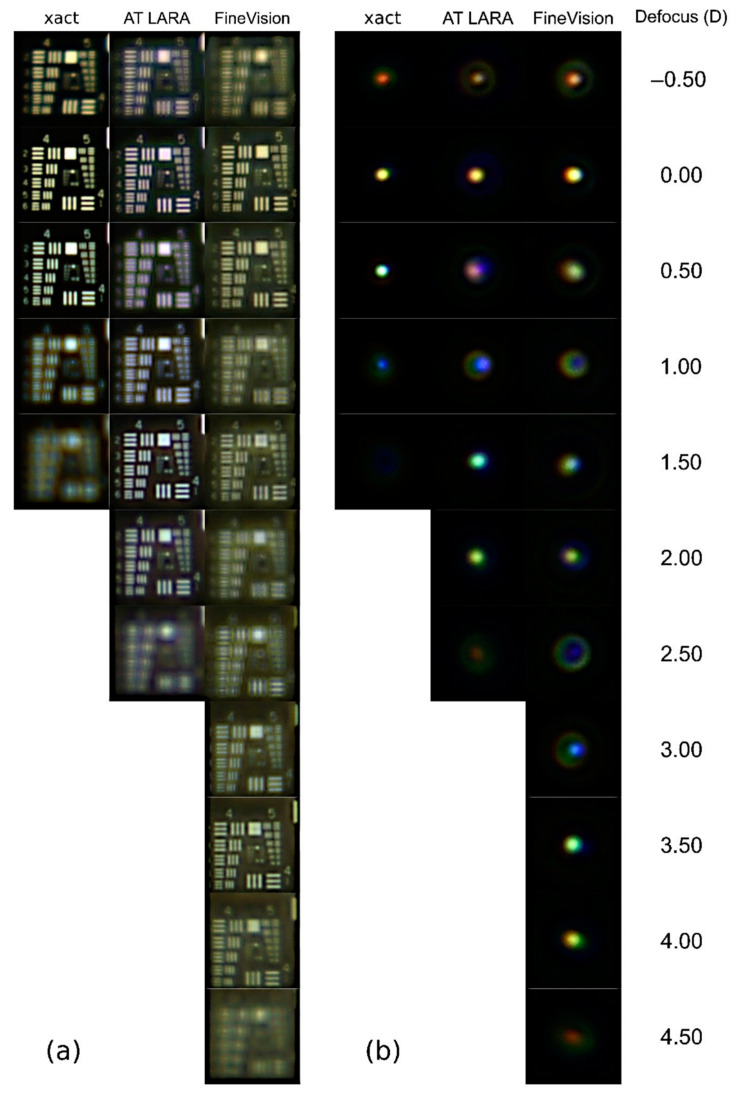
Through-focus images of the studied IOLs for the (**a**) USAF test and (**b**) a 30 µm pinhole as object tests.

Figure 2 shows the results of the USAF test images and their corresponding PSFs obtained with the three IOLs.

As can be seen in Figure 2a, the good vision ranges correspond to the characteristics of each design. In fact, the xact and AT LARA lenses produce a sharp vision range of approximately 1.00 D and 2.00 D, respectively. In the case of the AT LARA IOL, a loss of visibility in the focal plane of +0.50 D is evident. The FineVision images clearly show its trifocal behavior (although the plane of the intermediate focus, 1.75 D, is not shown because the sampling interval chosen is in steps of 0.50 D for succinctness). Although in Figure 2a it can be seen how the different diffractive designs affect the contrast of the images in each plane, the chromatic effects for each design are not very evident, except for the yellowish background in the images of the xact and FineVision IOLs. Note that this background is not the same for both lenses, indicating that the blue filters of these IOLs have a different bandwidth.

The influence of chromatic aberration and the haloes caused by each design are more evident in the PSFs with polychromatic light shown in Figure 2b. For the xact lens, it can be observed that the PSFs have high intensities, but with a hue ranging from orange (at −0.5 D) to blue (at +1.0 D). At the +1.00 D, where blue predominates, the extent and intensity of the PSF is the lowest due to the blue filter of the IOL. Regarding the dysphotopic phenomena that could be generated by this IOL when implanted, they would be very low, since the haloes in the focal range are of very low intensity.

The PSF provided by the AT LARA, at the far focus shows has an orange hue generated mainly by the contribution of red and green wavelengths; in fact, despite not having a yellow filter, the contribution of blue wavelengths to the far focus in this IOLs is negligible. However, for intermediate viewing planes, the contribution of blue increases and the PSF changes from purple to blue. Finally, the green wavelength generates a focus extension between 1.50 D and 2.00 D. The halos generated by this IOL are more noticeable at −0.50 D and 1.00 D vergences, yet they are still of low intensity.

The TF-PSFs of the FineVision lens show a different chromatic composition. In the intermediate vision range, between 1.5 and 2.0 D, the chromatic aberration is compensated. In the near focus, the predominance of blue at +3.0 D and red at +4.5 D indicate the presence of longitudinal chromatic aberration of diffractive origin. On the other hand, the PSFs around the far focus show a longitudinal chromatic aberration of opposite sign, indicating a predominance of refractive effects. In addition, in the defocused planes between the main foci (1.00, 2.00, 2.50 and 3.00 D) the haloes are wider as a consequence of the chromatic aberration.

### 3.2. Polychromatic TF-MTF

**Figure 3:**
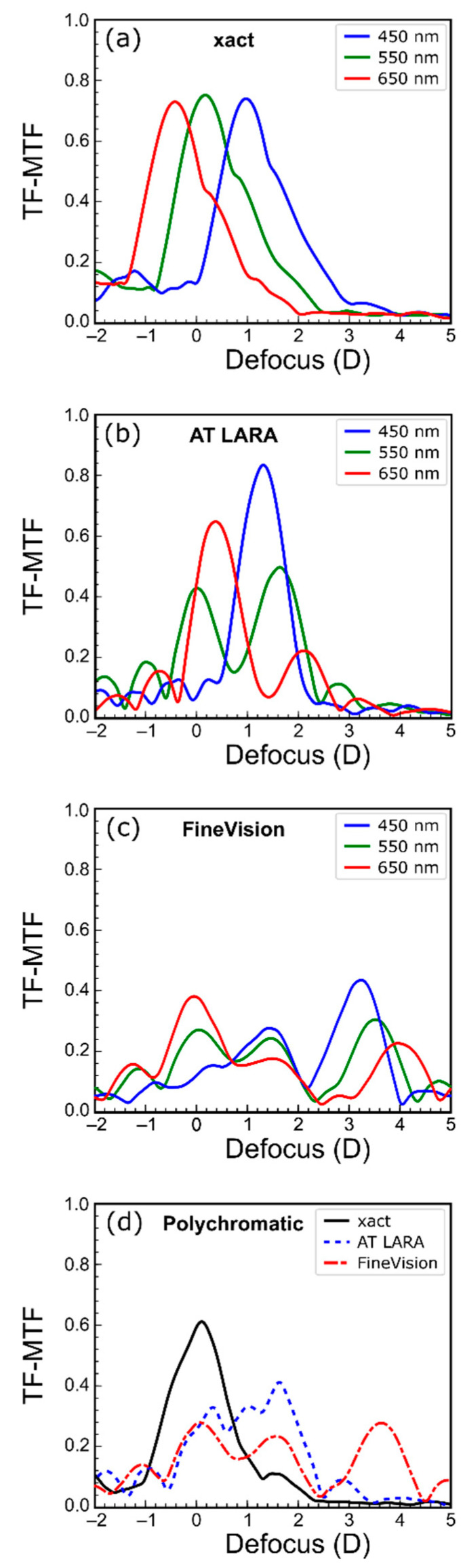
Experimental TF-MTFs obtained for the (**a**) xact, (**b**) AT LARA, and (**c**) FineVision for different wavelength monochromatic illuminations (450 nm, 550 nm, and 650 nm), and (**d**) for the three IOLs with polychromatic illumination.

Figure 3 shows TF-MTFs obtained with three chromatic filters (450 nm, 550 nm and 650 nm) and with polychromatic light. 

The TF-MTFs of the xact IOL are shown in Figure 3a. A wide asymmetric focus can be seen for all three wavelengths. This asymmetry, could be the result of an additional diffractive focus (i.e., due to the 4 diffractive rings in the central 3.00 mm) partially overlapping the refractive focus and producing an EDOF effect. Moreover, this asymmetry, although similar, is not exactly the same for all three wavelengths, which could be another consequence of the diffraction by the rings. However, the order in which the foci appear indicates that the dominant chromatic in this lens is due to refraction. The LCA measured in this lens is 1.41 D. At this point, it is important to remember that the MTFs were obtained as the contrast of the images in each plane and therefore the relative contrast values for each wavelength cannot be extrapolated to the intensity values at the foci shown in Figure 2. Therefore, in Figure 3, the contrast of the images with blue light can be very high at the foci, although, the relative intensity of the test image is very low, compared with the other wavelengths.

The TF-MTFs of the AT LARA (Figure 3b) show a very different profile for each wavelength. For green light, the IOL provides a bifocal profile of 1.80 D addition. However, for red and especially for blue, the profiles are more similar to that of a monofocal lens with maximum values at 0.4 D and 1.8 D, respectively. 

These two peaks are located between the foci generated by the green light, producing an extended in the depth of focus as shown in Figure 3d. In this case it is important to note that a correct evaluation of the LCA in this IOL is not possible due to the large differences in red, green, and blue TF-MTFs [27].

Figure 3c shows the trifocal profile of the FineVision lens for the different wavelengths. In green, there is one focus centered at 0.00 D, another at approximately 1.75 D and the third centered at 3.50 D. The LCAs of these foci are −0.40 D, 0.02 D and 0.80 D, respectively.

In Figure 3d, the TF-MTFs of the three IOLs obtained with polychromatic light are compared. The enhanced monofocal character of the xact lens, the EDOF effect character of the AT LARA and the trifocal profile of the FineVision are clearly visible in this figure. These results are in agreement with those shown in Figure 2a. The IOL xact has higher contrast but a smaller range of clear images compared to the other two lenses. The AT LARA IOL shows a lower contrast at distance but a higher contrast at intermediate distances, and a higher depth of focus. The polychromatic TF-MTF of the FineVision has three main foci with lower contrast than the other two IOLs, the contrast of this IOL is slightly better in near vision.

## 4. Discussion

Currently, there is a broad consensus among ophthalmologists that, when prescribing an IOL for presbyopia correction, it is very important to take into account the choice of the model that best suits the visual needs of each patient. In the performance of each IOL, the chromatic aberration plays a very important role. In fact, this is a parameter that some manufacturers take into account in their new premium IOL designs. However, the chromatic performance of these IOLs on an optical bench is still understudied. In addition, in the few studies carried out with polychromatic light, different instruments and metrics are used, which makes it difficult to establish comparative analyses. In order to provide more information that is relevant for surgeons in their decision making, in this work, we have measured the chromatic performance of three premium diffractive lenses with different designs under the same experimental conditions. To minimize the influence of SA, all measurements have been made for a 3.00 mm pupil.

According to our results, in the enhanced monofocal xact Mono-EDOF ME4, the chromatic aberration of the lens and the diffractive design extends the depth of focus. This IOL also stands out for the high contrast of the images obtained in distance vision and the low incidence of halos (Figure 2) compared to the other two IOLs. To our knowledge, this is the first study to assess the chromatic performance of the xact Mono-EDOF IOL. Previous optical bench studies of the xact were performed with monochromatic light (545 nm) [14,15]. Ruiz-Alcocer et al. [14] found that the TF-MTFs has two foci at 0.00 D and 1.00 D with a significant overlap. In our results for 550 nm (Figure 3a), both foci can also be observed at the same vergences; however, they are wider than those reported in Ref. [14]. This difference can be attributable to the different pupil diameters employed in both studies (3.00 mm and 4.50 mm). On the other hand, our results are also in agreement with clinical studies [12,13] performed with this IOL since they show that patients achieve a depth of focus in monocular vision of approximately 1.25 D. They also report a low incidence of dysphotopic phenomena [12,13], a result that is in line with our results shown in Figure 2b, where the absence of halos for this IOL stands out.

The EDOF effect AT LARA 829MP IOL was previously studied with different commercial instruments using monochromatic light (546 nm) [8,28]. In these works, images of the USAF test were obtained showing a depth of focus of 2.00 D, although both studies evidenced the bifocal behavior of the lens. This effect is also observed in the results obtained in this work, both in the USAF test images with polychromatic light (Figure 2a) and in the TF-MTF for 550 nm wavelength (Figure 3b). On the other hand, Łabuz et al. [9] performed a chromatic study with the AT LARA lens in which they used a commercial optical bench. The results they obtained were measured at wavelengths of 480 nm, 546 nm, 644 nm. For the TF-MTFs, different profiles are observed as a function of wavelength, which qualitatively coincide with the curves obtained in this work. Specifically, for the 546 nm wavelength the TF-MTF profile is bifocal, while for 480 nm and 644 nm, the profile resembles a monofocal behavior focusing on intermediate and distance vision, respectively. The major difference with our results of Figure 3b lies in the fact that its red focus coincides with the distance vision green focus and the blue focus coincides with the intermediate vision green focus, whereas our blue and red foci are located just between the two green foci, extending in this way the depth of focus of the IOL. This difference can be attributed mainly to two factors: First, in our study the LCA of the optical bench is offset, while in their study is 1.04 D; second, the reference wavelengths employed to obtain the LCA in both experiments are different, especially for the blue wavelength (the difference is 30 nm). Regarding the images obtained with the USAF test, Łabuz et al. [9] do not show intermediate plane images, but in the distance and near foci their results present a red and blue hue, respectively, as happens in our Figure 2a. At this point, it is important to note that the chromatic behavior of the AT LARA is similar to that of the TECNICS Symfony lens, also classified as “EDOF effect” [3,19].

The FineVision POD F trifocal IOL has been extensively studied both on an optical bench and in clinical trials [4,5,6,18,29]. From the studies with polychromatic light we highlight the work of Loicq et al. [18] in which measurements were taken with a commercial optical bench for a 3.00 mm pupil and wavelengths of 480 nm, 546 nm and 650 nm. In their results for the TF-MTF, the trifocal profile of this IOL is maintained for the three wavelengths and a LCA of opposite sign is observed in the distance and near foci, being higher (in absolute value) for the near focus. At the intermediate focus, the LCA is nearly zero [18]. Our results also show the trifocal profile of the lens and a similar LCA ratio between the foci, although the foci are slightly wider, and the intermediate focus is closer to the far focus. These small discrepancies between our results and those of Lociq et al. [18] may be due to the differences between both optical setups. In particular, small differences of the actual pupil diameter. This hypothesis is based on the results obtained by Rampat, and Gatinel [3] on this IOL with the same instrument, but with 2.00 mm pupil. The results in that case show two wider and partially overlapping TF-MTF peaks, corresponding to distance and intermediate vision, approaching to results we found in this study.

Finally, we should mention that this work is not free of limitations. On the one hand, we have used lenses of different base power. However, in our optical bench, this effect has a minimal influence because, thanks to the Badal system, the differences in the relative magnification of the images obtained with different power lenses is minimal. On the other hand, our study was limited to three models of different designs of premium IOLs, but all of them having a diffractive profile. Finally, since the main objective was to isolate the influence of chromatic aberration in each design, only the spherical aberration free ISO Model 1 cornea was used. Therefore, it is important to remark that optical bench results cannot be extrapolated to clinical outcomes, where the IOL is certainly the most important variable, but the cornea and its aberrations as well as pupil size and axial length also influence the outcomes.

## 5. Conclusions

In this work, we have shown how different optical designs of hybrid refractive–diffractive IOLs for presbyopia affect their chromatic behavior. On the one hand, the xact IOL benefits from its high chromatic aberration of refractive nature and its diffractive profile with a reduced number of rings to increase the depth of focus. In this regard, it is important to note that this is the first report of the xact IOL performance in an optical bench with polychromatic light. On the other hand, we have found that the AT LARA is designed so that each wavelength behaves differently but it produces an EDOF profile for polychromatic light. The results obtained for the polychromatic PSFs and the white light USAF images provided by this IOL at different defocused planes have not been previously reported and provide very important information, complementary to the polychromatic TF-MTF, to understand the performance of this EDOF effect IOL. Finally, as expected, the FineVision IOL, with its hybrid diffractive refractive design, minimizes chromatic aberration at the intermediate focus, but has positive LCA at the far focus and negative LCA at the near focus.

In summary, images of the USAF test at different planes under white light illumination test give a rough idea of the range of sharp vision and the relative contrast of the images. However, polychromatic PSF images at the same planes help to understand the relative contribution of each focus to the image intensity. Finally, both monochromatic and polychromatic TF-MTFs give information about the contrast of the images at each vergence. These figures are very useful to understand the optical principle of each lens and could be taken into account to predict clinical outcomes. Therefore, from our point of view, this information could help the clinician in choosing the lens that best suits the requirements of the individual patient.

## Figures and Tables

**Table 1:** Main specifications of the three IOLs included in this study.

	xact Mono-EDOF ME4	AT LARA 829MP	FineVision POD F
Optic design	Diffractive, aspheric	Diffractive, aspheric	Diffractive, aspheric
Base power (D)	+30.00	+14.00	+13.00
Material	Hydrophobic. UV and blue-light blocker	Hydrophilic (25%) acrylic with hydrophobic surface. UV blocker	Hydrophilic (26%) acrylic. UV and blue-light blocker
Body design	Single-piece/C-loop	Single-piece/plate-haptic/square edge	Single-piece/double C-loop
Optical/total diameter (mm)	6.00/12.50	6.00/11.00	6.00/11.40
Spherical aberration (µm)	−0.17	0.00	−0.11
Refractive Index	1.54	1.46	1.46
Abbe Number	N.A.	56.50	58.00

## Data Availability

The data that support the findings of this study are available from the corresponding author, W.D.F., upon reasonable request.
